# Establishment of a Sonotrode Ultrasound-Assisted Extraction of Phenolic Compounds from Apple Pomace

**DOI:** 10.3390/foods11233809

**Published:** 2022-11-25

**Authors:** María del Carmen Razola-Díaz, María José Aznar-Ramos, Eduardo Jesús Guerra-Hernández, Belén García-Villanova, Ana María Gómez-Caravaca, Vito Verardo

**Affiliations:** 1 Department of Nutrition and Food Science, Campus of Cartuja, University of Granada, 18011 Granada, Spain; 2 Institute of Nutrition and Food Technology ‘José Mataix’, Biomedical Research Centre, University of Granada, Avda del Conocimiento sn., 18100 Armilla, Spain; 3 Department of Analytical Chemistry, University of Granada, Campus of Fuentenueva, 18071 Granada, Spain

**Keywords:** HPLC–MS, *Malus*, antioxidant activity, amygdalin, by-product, waste revalorization

## Abstract

Apple pomace is the main by-product from apple processing in the juice industry and is considered a source of polyphenols with several health bioactivities. Thus, this research focuses on the establishment of the ultrasound-assisted extraction of total phenolic compounds, focusing on phloretin and phloridzin, with high antioxidant activity from apple pomace, using a sonotrode. We used a Box–Behnken design of 15 experiments with 3 independent factors (ethanol (%), time (min) and amplitude (%)). The responses evaluated were the sum of phenolic compounds, phloretin and phloridzin measured by HPLC–MS-ESI-TOF, and antioxidant activity measured by DPPH, ABTS and FRAP. The validity of the model was confirmed by ANOVA. Further, it was carried out using a comparison between different apple pomaces with or without seeds extracted by the optimal conditions. Phloretin and phloridzin accounted for 7 to 32% of the total phenolic compounds in the apple pomaces. Among all the apple pomace analyzed, that of the variety Gala had the highest phenolic content and antioxidant activity. The presence of the cyanogenic compound amygdalin was detected in apple pomaces that contained seeds accompanied with a higher content of phloretin and phloridzin but a lower content of flavan-3-ols.

## 1. Introduction

Bioprospecting to recover wastes of natural origins is a very tangible proposal in different sectors, from the agronomic [1,2] to forest management [3] as well as for marine by-product valorization [4] to address a transition from a linear to a circular economy model. In this framework, apple is the edible fruit of the species *Malus domestica* named by Moritz Balthasar Borkhausen in 1803, the common apple tree. *Malus domestica* is a pome fruit with a round shape and a very sweet flavor, depending on the variety. The worldwide apple production in 2020 accounted for 86.44 million metric tons, 17.7% higher than in 2010, and the tendency is clearly increasing. In 2020/2021, the major producer of apples worldwide was China (54.7%) followed by the European Union (14.6%), the United States (5.6%) and Turkey (5.3%). Apple juice was the most produced juice in 2017 with a market share of 15.7%. After Germany, Poland, the United Kingdom and France, Spain was the fifth most producer of apple juice with a production of 103.475 mil litters. Further, in the European Union, 2.04 million litters were produced in 2017, 17.7% higher than in 2008 [5]. However, from this juice industry, they generated a huge number of by-products known as apple pomace. According to Shalini et al. [6], these by-product account for 25% of the processed apples. Apple pomace has been reported to have several bioactivities such as prebiotic, hypo-cholesterolemic, antioxidant, antimicrobial, anti-inflammatory, anticancer, and cardio-protective effects [7]. In recent years, the revalorization of this apple pomace by-product has been investigated for different purposes—as beer flavoring [8], in dermal formulations [9], as natural fillers in polymeric composites [10], as a fortification ingredient in meat products [11], and for biofuel production [12], among others [7]. Further, apple pomace is a source of phenolic compounds such as dihydrochalcones, flavan-3-ols, flavonols, anthocyanins, and hydroxycinnamic acids [13]. The revalorization of the phenolic compounds present in the apple pomace has been more complicated because of the presence of the degrading enzyme polyphenol oxidase in combination to its high moisture (≥80%) and sugar content. Some authors are in search of the best procedure to deal with it and blanching has been discovered to be enough for inactivating polyphenol oxidase activity in apples. However, as reported by Heras-Ramírez et al. [14], the drying of blanched or unblanched apple pomace causes a significant reduction in bioactive phenolic compounds. Otherwise, according to Yan et al. [15], vacuum freeze drying is a good alternative to produce apple pomace powders without losing phenolic content, anthocyanins and dietary fiber, but it is expensive and is not affordable to apple processing factories. Thus, a technology that processes the apple pomace immediately after its generation can provide the solution to industries facing economic loss on its disposal [13]. Some authors have studied the extraction of polyphenols from apple pomace with different techniques [16] such as thermal maceration [17,18], assisted with enzymes [19], assisted with non-ionic emulsifiers [20], microwave-assisted extraction [21,22,23,24], and supercritical fluid extraction [25,26]. Although there is little research about the use of ultrasound technology for extracting phenolic compounds [27,28], it has been applied previously to apple pomace with other aims such as isolating xyloglucans [29], or extracting pectin [30]. Further, ultrasound technology for optimizing the extraction of bioactive compounds has been performed by other authors in other matrices such as eggplant [31], orange peel [32], tangerine [33], cashew apple bagasse [34] or onion leaves [35].

Thus, the aim of this work was the establishment of optimized ultrasonic-assisted extraction by sonotrode using a Box–Behnken design to obtain the highest phenolic content, especially phloretin and phloridzin, from apple pomace and the highest in vitro antioxidant activity measured by DPPH (2,2-diphenyl-1-picrylhydrazyl), FRAP (ferric reducing antioxidant power) and ABTS (2,2′-azino-bis-3-ethylbenzothiazoline-6-sulphonic acid) assays. For that purpose, the determination of phenolic compounds by using HPLC–MS was carried out. In addition, different apple pomaces obtained from different apple varieties were compared and the presence of amygdalin was also evaluated.

## 2. Materials and Methods

### 2.1. Chemicals

Double-deionized water used in the analysis was obtained with a Milli-Q system (Millipore, Bedford, MA, USA). DPPH, ABTS, potassium persulfate, 6-hydroxy-2,5,7,8-tetramethylchroman-2-carboxylicacid (Trolox) and 2,4,6-Tris(2-pyridyl)-s-triazine (TPTZ) were supplied by Sigma-Aldrich (St. Louis, MO, USA). Ethanol, methanol, and hydrochloric acid were provided by Panreac (Barcelona, Spain). Standards vanillic acid, chlorogenic acid, ferulic acid, quercetin, catechin, phloretin, phloridzin, amygdalin and rutin were also purchased from Sigma-Aldrich (St. Louis, MO, USA). Other reagents were purchased from Merck KGaA (Darmstadt, Germany).

### 2.2. Samples

Juices from apples (*Malus*) from the varieties *M. pumila* cultivar ‘Fuji’, *M. domestica* cultivar ‘Golden Delicious’, *M. domestica* cultivar ‘Gala’ and *M. domestica × M. sylvestris* cultivar ‘Granny Smith’ were obtained by a manual press (30162, WilTec Wildanger Technik GmbH, Eschweiler, Germany) with a press chamber volume of 6 L and a 19.5 cm diameter and 26 cm height. After filling the press with 1 kg of apple, inside a polyester mesh (30358, WilTec Wildanger Technik GmbH, Germany), the samples were pressed till all the juice was extracted. The percentage of seed in those apple pomaces was approximately 1–1.5% of total apple pomace. Another trial was obtained eliminating the seeds from the apples before the pressing. Then, the remaining apple pomaces were collected (with and without seeds), the moistures were measured (78–82%), and the polyphenol extractions were performed directly in triplicate in fresh samples. All the pressing process was carried out in less than 30 min trying to avoid the degradation of phenolic compounds.

### 2.3. Experimental Design

A Box–Behnken design combined with response surface methodology (RSM) was carried out to optimize the conditions of extracting phenolic compounds with high antioxidant activity from the apple by-product via ultrasound-assisted extraction with sonotrode. For the modelling, apple pomace mix was used from the different varieties without seeds. The experimental model was composed of 15 experiment structures in three blocks with three levels (−1, 0, +1) corresponding to a lower, intermediate and a higher value for each parameter. Each experiment was carried out in duplicate. The independent variables had into account were ethanol (0, 50, 100%), time (5, 25, 45 min) and amplitude (20, 60, 100%). The responses analyzed were the content of phloretin, phloridzin and the sum of phenolic compounds analyzed by HPLC–MS, and the antioxidant activity measured by DPPH, ABTS and FRAP. These dependent variables were adjusted to a second-order polynomial model equation (Equation (1)), where Υ represents the response variable, X_i_ and X_j_ are the independent factors that affect the response, and β_0_, β_i_, β_ii_ and β_ij_ are the regression coefficients of the model (interception, linear, quadratic and interaction terms). Statistica 7.0 package (StatSoft, Tulsa, OK, USA) was used for the mathematical operations and simulations.

Equation (1). Second-order polynomial equation.

(1) $$\Upsilon ={\;\beta }_{0}+\overset{4}{\underset{i=0}{\sum }}\beta_{i}X_{i}+\overset{4}{\underset{i=0}{\sum }}\beta_{\mathrm{ii}}X_{\mathrm{ii}}^{2}+\overset{4}{\underset{i=0}{\sum }}\overset{4}{\underset{j=0}{\sum }}\beta_{\mathrm{ii}}X_{i}X_{j}$$

Additionally, ANOVA was performed to evaluate the adjustment of the model having into account the regression coefficients, the *p*-values of the regressions and the lack of fit. Optimum conditions were established using RSM through three-dimensional graphs of the responses. Further, the optimal conditions were validated and confirmed.

### 2.4. Ultrasound-Assisted Extraction by Sonotrode

Briefly, an amount of 6 g fresh apple pomace (with or without seed) was extracted with 100 mL of an ethanol/water solution by a sonotrode (UP400St ultrasonic processor, Hielscher, Germany) with the probe S24d14D according to the conditions established in the model. Temperature was not controlled. After the extraction, the samples were centrifuged at 9000 rpm for 10 min. Then, the supernatant was collected, evaporated by rotavapor and the extract was reconstituted in 1 mL of methanol/water 1:1 (*v*/*v*). Finally, it was stored at −18 °C until the analyses.

### 2.5. Antioxidant Assays

DPPH, ABTS and FRAP assays were carried out to determine the antioxidant capacity of the apple pomace extracts by the procedures described in previous research [32,36,37]. In all assays, Trolox was used as the standard for the calibration curves and the results were expressed in mg of Trolox equivalents (TE)/g of dry weight (d.w.). The measurements were performed using an UV–visible spectrophotometer (Spectrophotometer 300 Array, UV–Vis, single beam, Shi-madzu, Duisburg, Germany).

### 2.6. Determination of Phenolic Compounds by HPLC–ESI–TOF–MS Analysis

Phenolic compounds present in the apple pomace extracts were analyzed using an Acquity Ultra Performance Liquid Chromatography (UPLC) system (Waters Corporation, Milford, MA, USA) coupled to an electrospray ionization (ESI) source operating in the negative-mode and a mass detector time of flight (TOF) micro mass spectrometer (Waters). The compounds of interest were separated on an ACQUITY UPLC BEH Shield RP18 column (1.7 µm, 2.1 × 100 mm; Waters Corporation, Milford, MA, USA) at 40 °C using the conditions and gradient previously used by Verni et al. [38]. H_2_O acidified with 1% of acetic acid and acetonitrile were used as phase A and B, respectively. Analyses were performed in triplicate.

**Figure 1:**
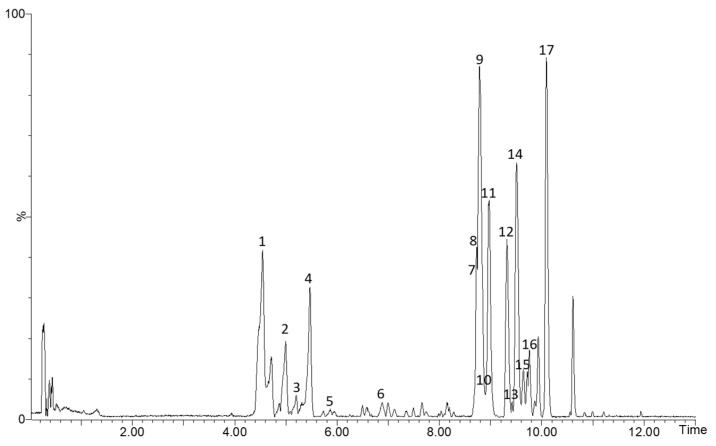
HPLC–TOF–MS chromatograms of apple pomace without seed extracts. 1: caffeoylquinic acid; 2: catechin; 3: epicatechin; 4: coumaroylquinic acid; 5: procyanidin dimer; 6: procyanidin trimer; 7: phloretin; 8: phloretin-2′-O-xyloglucoside; 9: quercetin-3-O-galactoside; 10: rutin; 11: quercetin-3-O-glucoside; 12: phloridzin; 13: isorhamnetin-3-O-glucoside; 14: quercetin-3-O-arabinopyranoside; 15: quercetin-3-O-arabinofuranoside; 16: quercetin-3-O-xylanoside; 17: quercetin-3-O-rhamnoside.

MassLynx 4.1 software (Waters Corporation, Milford, MA, USA) was used for elaborating the data. The identification of the phenolic compounds was made according to literature. An indicative base peak total ion chromatogram of the apple pomace samples analyzed by HPLC–MS is shown in Figure 1. All the identified compounds are described in Table 1, with their retention time (min), molecular formula, experimental and calculated *m*/*z*, score (%), error (ppm) and in source *m*/*z* fragments. For ensuring the mass accuracy, the tolerances chosen had a score higher than 90% and error lower than 5 ppm. To quantify the phenolic compounds identified in apple pomace extracts, nine calibration curves were calculated: amygdalin, vanillic acid, chlorogenic acid, ferulic acid, quercetin, catechin, phloretin, phloridzin and rutin, in the range of 5–250 µg/mL.

## 3. Results and Discussion

### 3.1. Identification of Phenolic Compounds by HPLC–ESI–TOF–MS

The extracts of apple pomace without seed were analyzed by HPLC–ESI–TOF–MS and the identified compounds are presented in Table 1. A total of 17 phenolic compounds were found.

Two phenolic acids were identified at 4.56 and 5.31 min as caffeoylquinic acid and coumaroylquinic acid, respectively, in concordance with other authors [17,22]. In addition, flavan-3-ols is a well-known group of flavonoids extensively found in apple matrices. So, according to previous studies [17,21,22,39,40,41], they were identified catechin, epicatechin, procyanidin dimer and procyanidin trimer corresponding to peaks 2, 3, 5 and 6, respectively. Three chalcones were found with *m*/*z* 273, 567 and 435, phloretin, phloretin-2-O-xyloglucoside and phloridzin, respectively. These compounds have been previously reported to be in apple pomaces by several authors [17,21,22,39,40,41,42]. Further, with the molecular formula C_22_H_22_O_12_, an isorhamnetin derivative was detected, isorhamnetin-3-O-glucoside according to Çam et al. [17]. Moreover, the flavonoid glycoside rutin was identified with *m*/*z* 609 at 8.84 min [17,22,39,40]. Finally, corresponding to peaks 9, 11, 14–17, six quercetin derivatives were detected according with *m*/*z* fragments 300–301—quercetin-3-O-galactoside, quercetin-3-O-glucoside, quercetin-3-O-arabinopyranoside, quercetin-3-O-arabinofuranoside, quercetin-3-O-xylanoside and quercetin-3-O-rhamnoside—in agreement with other authors [17,40,42]. Figure 1 shows a representative chromatogram of all the identified compounds in apple pomace without seed extracts.

### 3.2. Fitting the Model

This was carried out using a Box–Behnken design to optimize % of ethanol (X_1_), time (X_2_) and amplitude (X_3_) in ultrasound-assisted extraction by sonotrode to extract phenolic compounds with high antioxidant activity from apple pomace. Further, the responses evaluated were the content in phloretin, phloridzin, the sum of phenolic compounds and the antioxidant activity measured by three methods—DPPH, ABTS and FRAP—and the results for each run are presented in Table 2.

As can be seen in Table 2, in all cases, ultrasound technology enabled the release of the targeted phenolic compounds. Apple pomace is a vegetable tissue composed of vegetable cells with multiple layers of thick cellulose cell wall being more difficult to lyse than animal cells. Via this ultrasound treatment, the cavitation caused inside the cells enhanced the diffusion of phenolic and antioxidant compounds across the cell walls or provoked the rupture of the cells, releasing all the content to the extraction solvent. For phloretin and phloridzin, the obtained results ranges were 1.2–13.0 and 5.9–67.5 µg/g d.w., respectively. In both cases, the lowest recoveries were obtained when using 0% ethanol at the lowest time (5 min) or lowest amplitude (20%). The sum of phenolic compounds content was between 462.23 and 1878.74 µg/g d.w. In this case, lower recoveries were found when using 0% ethanol, but the lowest recovery was when treating during 5 min at the maximum amplitude (100%). Regarding the antioxidant activity, the results obtained were in the ranges of 0.61–2.79, 1.65–4.92 and 1.10–3.58 mg TE/g d.w. for DPPH, ABTS and FRAP, respectively. The lowest radical scavenging activity was found when using 100% ethanol in all the three methods. For all the evaluated variables, the highest recoveries were found at the intermediate conditions for the three independent factors (ethanol 50%, 25 min and amplitude 60%). This demonstrated that a mixture of water, a polar solvent, and ethanol, a less polar solvent is needed for extracting more phenolic compounds from apple pomace. Further, the highest amplitude and time seemed to increase the temperature of the samples, destroying not only the vegetable cell but also the interesting free phenolic compounds released. In contrast, the lowest time and amplitude were not enough for improving the extraction of polyphenols. Moreover, for all the evaluated responses, positive significant (*p* < 0.05) Pearson correlations with values from r = 0.4137 to r = 0.9627 were found.

Therefore, the data obtained experimentally were adjusted to a second-order polynomial equation, a regression model that provides the lowest residual value using the least-squares method. The regression coefficients of the model are presented in Table 3.

The model was analyzed with a significance level of *p* < 0.05. All the linear terms (β_1_, β_2_ and β_3_) and quadratic terms (β_11_, β_22_ and β_33_) showed a significant effect in all the response variables except the linear term amplitude (β_3_) in DPPH. Further, the three linear regression coefficients showed a positive effect among the responses except for β_1_, in the antioxidant assays. Regarding the crossed terms, all of them had significant effects for phloridzin and FRAP. Further, for phloretin the crossed between ethanol and time (β_12_) and for the sum of phenolic compounds and DPPH the crossed term between time and amplitude (β_23_) had significance. Additionally, the crossed effects β_13_ and β_23_ showed a significant effect for ABTS. After discarding the non-significant terms, the model was recalculated and tested by ANOVA. As can be seen in Table 3, the models revealed a high regression correlation between the dependent variables and the independent factors (R^2^ > 0.9585). Moreover, they all showed good fit to the regression model (*p* < 0.05), and no significant lack of fit (*p* > 0.05); therefore, as reported by Bezerra et al. [43], the adequacy of the model is confirmed.

**Figure 2:**
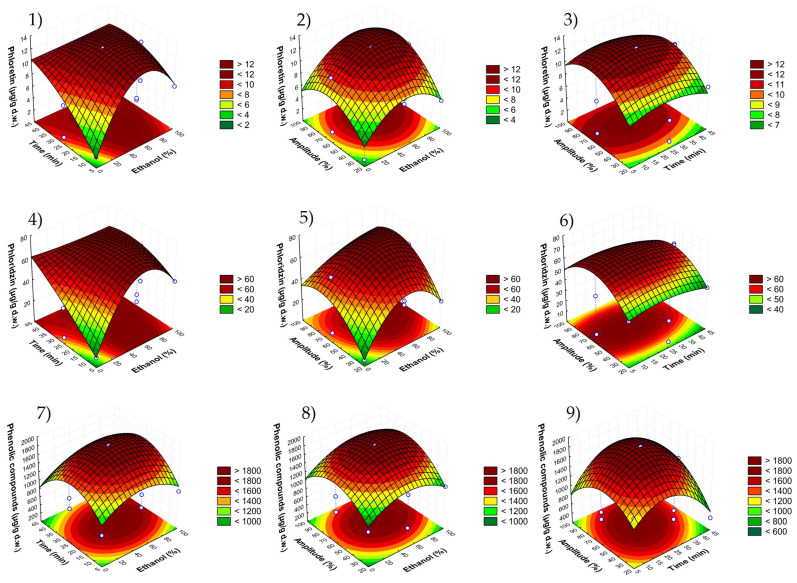
Response surface graphs (1–9) showing the combined effects of the process variables: ethanol (%), time (min) and amplitude (%) for the response variables 1–3: phloretin; 4–6: phloridzin; 7–9: sum of phenolic compounds.

**Figure 3:**
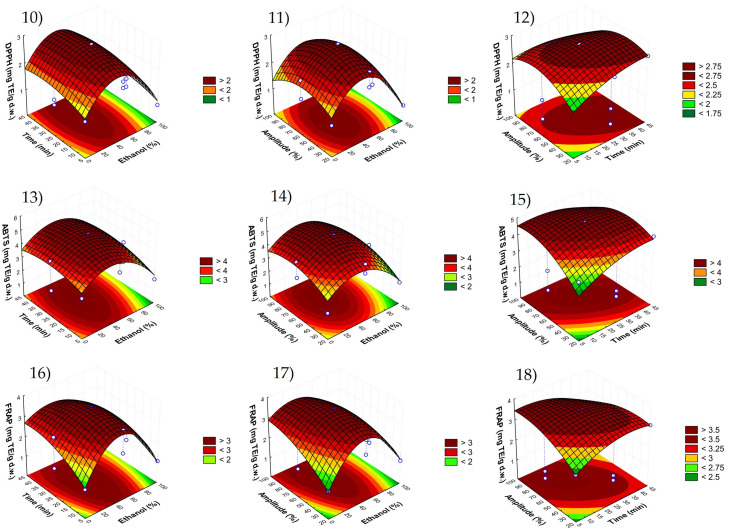
Response surface graphs (10–18) showing the combined effects of the process variables: ethanol (%), time (min) and amplitude (%) for the response variables 10–12: DPPH; 13–15: ABTS; 16–18: FRAP.

The optimal conditions were selected using response surface methodology (RSM) among the three-dimensional graphs shown in Figure 2 and Figure 3.

These figures showed the effects of the combination of the three independent factors in each response variable evaluated, phloretin (graphs 1–3), phloridzin (graphs 4–6), total phenolic compounds (graphs 7–9), DPPH (graphs 10–12), ABTS (graphs 13–15) and FRAP (graphs 16–18). Thus, there was a compromise between the minimum possible value of each independent factor to reach the maximum responses. As can be seen, ethanol percentages lower than 30% and higher than 60% lead to a reduction in all the dependent variables. Regarding the combined effect of time with amplitude, intermediate values of those two parameters allowed the highest recoveries. Therefore, the best conditions were established as 50% ethanol, 23 min and 65% amplitude, providing the predicted values shown in Table 4.

By using these conditions, the obtained values did not report significant differences (*p* < 0.05) with the predicted, with coefficients of variation lower than 5% in all the cases. So, the validity of model was confirmed. Further, these results are in the same range of magnitude as the data reported by other authors [25,44]. Derakhshan et al. [27] optimized the ultrasound extraction conditions from apple pomace with ethanol 70%, finding 82.36% amplitude, 35.24 min and 51.48 ºC as optimal conditions, obtaining a result of 74.53 mg GAE/100 g. Egües et al. [28] also tried to find the best conditions for extracting phenolic compounds from apple pomace by ultrasound technology using water as the solvent. They found 20 min, 90 °C and 50% amplitude as the best conditions, giving an optimum predicted value of phenolic compound of 6.07 mg GAE/g. In both cases, they used a spectrophotometric measurement (the Folin–Ciocalteu method) for the total phenolic content not having into account the recovery of phloridzin or phloretin or other specific phenolic compounds. Further, they both used high temperatures and it is well known that phenolic compounds such as anthocyanins and flavonols as quercetin, rutin, catechin and its derivatives are highly thermolabile [45,46]. Pollini et al. [47] compared different non-conventional extraction techniques such as ultrasound-assisted extraction, ultraturrax extraction, accelerated solvent extraction and pulsed electric field extraction pre-treatment to isolate phenolic compounds and especially phloridzin from red delicious apple pomace. The overall best ethanol concentration was 50%. However, for the ultrasound-assisted extraction, they used a temperature of 60 °C during 60 min, obtaining a phloridzin content of 71.19 µg/g in fresh apple pomace. In this work, the authors agreed with them and found a mixture of ethanol/water 50:50 as the optimum solvent. Moreover, the optimal time was lower than the previous studies (23 min) and the amplitude (65%) found here is in the range reported by them. Further, all the previous studies used an ultrasonic bath for the ultrasound-assisted extraction and the extraction optimized with sonotrode technology so that it could be easily scalable and allowed us to obtain extracts with the highest recoveries of phloretin, phloridzin and the sum of phenolic compounds with high antioxidant activity.

### 3.3. Comparison of Amygdalin and Phenolic Content in Different Samples

The phenolic compound profile and the antioxidant activity (DPPH, ABTS and FRAP) of different apple pomaces obtained from different apples are collected in Table 5. Further, the amygdalin content was also analyzed. This was identified in the negative ion mode with *m*/*z* 456, molecular formula C_20_H_26_NO_11_, and its main product ion 323 as shown in Table S1, in concordance with other authors that previously identified amygdalin [48,49,50]. Further, the negative-mode product ion chromatogram of amygdalin is shown in Figure S1. According to Lee et al. (2013) [48], the fragment ion 323 corresponds to amygdalin losing the disaccharide, as well as fragment ions of *m*/*z* 221 and 263 that were identified corresponding to the cross-ring bond cleavage of glucose A_2_. Additionally, the linkage of amygdalin with Cl^−^ in concordance with Guć et al. (2020) can be appreciated in the fragment ion *m*/*z* 492 [49]. In addition, Figure S2 shows the HPLC–ESI–TOF–MS chromatogram of apple pomace with seed extracts, showing the location of amygdalin at time 3.47 min.

As can be seen, the total phenolic content of the apple pomaces analyzed ranged from 922.39 to 1724.47 µg/g d.w., with the highest in Gala and the lowest in Granny. Further, the total flavan-3-ol content was in the range 15.4–46.8% of the total phenolic compounds, with the highest in Granny without seed. For the total quercetin derivatives, the results were in the range 8.4–56.3% of the total phenolic compounds, with the highest in the case of Granny and Fuji with seed. In the case of the sum of phloretin and phloridzin content, they accounted for 7.3–32.2% of the total phenolic compounds in the apple pomaces, with the highest in Golden with seed. In all cases, the presence of seeds in the pomace accounted for a decrease in the flavan-3-ol content (6–25%) and an increase in the quercetin derivatives (7.5–34%) and phloretin and phloridzin content (up to 13%). Cetkovic et al. [39], comparing different apple pomace from different apple varieties, reported the results of total phenolics from 0.69 to 1.47 mg/g d.w. measured by HPLC–DAD. Further, they reported ranges of 0.007–0.085, 0.02–0.13, 0.02–0.17, 0.21–0.48 and 0.29–0.61 mg/g d.w. for phloridzin, catechin, epicatechin, rutin and the sum of quercetin glycosides, respectively, in concordance with the results found here. It is a fact that the apple variety, harvesting date and environmental growth conditions affect the apple pomace phenolic composition. Apart from that, the differences found in respect to the results reported by other authors can be attributable to the different apple pomace precedence. As reported by Rabetafika et al. [51], the phenolic compound composition of the apple pomaces can be very different if obtained from the juice, cider, or syrup industry, and depending on if it is composed of the peel/skin and the seeds all together or separately.

Amygdalin is a cyanogenic glycoside naturally present in plant tissues such as in apples seeds. When amygdalin interacts with endogenous apple tissue digestive enzymes, hydrogen cyanide is released and is highly toxic, reported with symptoms such as headaches, dizziness, hypotension, loss of consciousness, coma, and death [52]. Previously, other authors have reported amygdalin contents in the range of 0.1–17.5 mg/g in apple seeds [53]. In this case, the contents of amygdalin ranged between 13.14 µg/g d.w. in Gala and 60.07 µg/g d.w. in Golden. A positive strong correlation between the content of phloretin and phloridzin and the presence of amygdalin (0.6683 and 0.6808, respectively) was found (Figure S3). This is in concordance with other authors, who also reported higher amounts of phloretin and phloridzin in the seeds than in other parts of the apple such as as the skin [54]. In contrast, the same kind of correlation but negative has been found between the flavan-3-ol and the amygdalin content. Further, a negative correlation between the content of phloretin and phloridzin and the content in total flavan-3-ols in these samples of apple pomace was discovered (−0.7754 and −0.7380, respectively). These results clearly indicate that higher concentrations of phloretin and phloridzin, lower in procyanidins and the presence of amygdalin could be used as markers of the presence of seeds in the apple pomaces. According to the CDC (Centres for Disease Control and Prevention), the revised IDLH (Immediately Dangerous to life or health concentrations) for cyanides is 25 mg CN/m^3^ based on acute oral toxicity data in humans [55]. Moreover, in 2017, the Commission Directive 2017/164/EU changed the indicative occupational exposure limit to hydrogen cyanide to 0.9 mg/kg over the long term and 4.5 mg/kg over the short term [56]. Considering that, according to Dang et al. [57], 500 mg of amygdalin could contain as much as 30 mg of cyanide, and that the maximum amount of amygdalin detected in the compared apple pomaces with seed was approximately 60 µg per g of apple pomace, for cyanide poisoning to occur in a person of 60 kg, it would be necessary to consume approximately 75 kg of apple pomace. In this context, it can be concluded that the presence of seeds that contain amygdalin in the apple pomaces is not a potential danger for health if used in low amounts taking into consideration the potential benefits of the increased phloretin and phloridzin content, etc.

For the antioxidant activity, all the three methods showed high significant (*p* < 0.05) correlation (r > 0.95) (Figure S3). Phloretin and phloridzin showed the highest significant (*p* < 0.05) positive correlation, with antioxidant activity values of 0.7476–0.8577 and 0.5639–0.7040, respectively. Further, quercetin derivatives showed a higher significant (*p* < 0.05) positive correlation with the antioxidant activity measured by the three methods (0.8960, 0.7323 and 0.7378 for DPPH, ABTS and FRAP, respectively). This is in concordance with the results obtained by other studies such as Diñeiro-García et al. [41], who analyzed cider apple pomaces reported to have highly significant correlations between the antioxidant activity and the content in phloridzin and total phenols and phloretin 2-xyloglucoside. Grigoras et al. [22] compared different apple pomace varieties through antioxidant activity by DPPH and the results classified them from highest to lowest antioxidant activity: Golden > Granny > Gala. In this study, the positions of Gala and Golden apple pomaces are inverted (Gala > Granny > Golden). Similarly, Persic et al. [58] reported higher total phenolic content in Granny than in Golden. Rana et al. [59] compared different apple pomaces from Royal Delicious, Red Delicious, Golden Delicious, Red Chief and Red Gold apples finding antioxidant activity results in the same range of magnitude obtained here. They also highlighted a varietal influence on phenolic composition.

## 4. Conclusions

A Box–Behnken design was used to establish the best parameters for ultrasound extraction by sonotrode for obtaining higher amounts of phloretin, phloridzin, the sum of phenolic compounds and antioxidant activity (DPPH, ABTS and FRAP) from apple pomace. The optimal sonotrode conditions selected were 50% ethanol, 23 min and 65% amplitude. The use of sonotrode extraction has been demonstrated to be a non-thermal, time-efficient and scalable method that allows the recovery of phloretin and phloridzin, among others, with a high content of antioxidants from apple pomace that could be used as functional ingredients. Further, juice apple pomaces with and without seeds from different varieties extracted by the optimal conditions were compared. All the extracts were characterized by HPLC–ESI–TOF–MS and 17 phenolic compounds were identified and quantified. Among all the varieties, Gala and Granny smith apples exhibited higher polyphenol content. Moreover, the presence of seeds in the apple pomace did not reveal a potential danger for health taking into consideration the potential benefits such as the increased content of phloretin, phloridzin and quercetin derivatives. For future research, it would be interesting to evaluate the in vivo antioxidant activity of the apple pomace extracts obtained by the optimized sonotrode conditions established and evaluate other potential activities for its application in terms of food, nutraceuticals, and cosmeceuticals.

## Figures and Tables

**Table 1:** Compounds identified by HPLC–ESI–TOF–MS in apple pomace without seed extracts.

Peak	Rt (min)	Observed *m*/*z*	Calculated *m*/*z*	Error (ppm)	Score (%)	Molecular Formula	In Source *m*/*z* Fragments	Compound Name
1	4.56	353.0863	353.0873	−2.8	98.15	C_16_H_18_O_9_	191.0532; 179.0342; 173.0427; 135.0425	Caffeoylquinic acid
2	4.99	289.0703	289.0712	−3.1	91.47	C_15_H_14_O_6_	245.0740; 125.0235	Catechin
3	5.20	289.0699	289.0712	−4.5	90.97	C_15_H_14_O_6_	125.0233	Epicatechin
4	5.31	337.0907	337.0923	−4.7	93.86	C_16_H_18_O_8_	173.0423; 191.0530; 235.0558	Coumaroylquinic acid
5	5.41	577.1357	577.1346	1.9	99.83	C_30_H_26_O_12_	407.007; 289.0711	Procyanidin dimer
6	6.88	865.2006	865.1980	3.0	99.71	C_45_H_38_O_18_	407.0749; 289.0721	Procyanidin trimer
7	8.72	273.0754	273.0763	−3.3	97.23	C_15_H_14_O_5_	167.0319	Phloretin
8	8.73	567.1715	567.1714	0.2	97.67	C_26_H_32_O_14_	273.0730; 167.0313	Phloretin-2′-O-xyloglucoside
9	8.78	463.0868	463.0877	−1.9	93.12	C_21_H_20_O_12_	301.0390; 271.0211; 241.0108	Quercetin-3-O-galactoside
10	8.84	609.1438	609.1456	−3.0	93.17	C_27_H_30_O_16_	463.0861; 301.0303	Rutin
11	8.97	463.0857	463.0877	−4.3	99.96	C_21_H_20_O_12_	301.0301; 271.0202; 241.0096	Quercetin-3-O-glucoside
12	9.31	435.1276	435.1291	−3.4	99.95	C_21_H_24_O_10_	273.0739; 167.0320	Phloridzin
13	9.33	477.1010	477.1033	−4.8	92.24	C_22_H_22_O_12_	315.0110; 287.0181; 331.0412	Isorhamnetin-3-O-glucoside
14	9.51	433.075	433.0771	−4.8	99.97	C_20_H_18_O_11_	301.0314; 271.0214; 241.0117	Quercetin-3-O-arabinopyranoside
15	9.64	433.0754	433.0771	−3.9	99.77	C_20_H_18_O_11_	300.0248; 271.0206; 241.0113	Quercetin-3-O-arabinofuranoside
16	9.72	433.0759	433.0771	−2.8	90.11	C_20_H_18_O_11_	301.0335; 271.0222; 241.0109	Quercetin-3-O-xylanoside
17	10.09	447.0921	447.0927	−1.3	97.64	C_21_H_20_O_11_	301.0330; 271.0236; 255.0279	Quercetin-3-O-rhamnoside

**Table 2:** Box–Behnken design with natural and coded values (parenthesis) of the conditions of extraction and the experimental results obtained for phloretin, phloridzin, the sum of phenolic compounds and antioxidant assays (DPPH, ABTS and FRAP) expressed as the average ± standard deviation.

Run	Independent Factors			Responses					
	X1	X2	X3	Phloretin (µg/g d.w.)	Phloridzin (µg/g d.w.)	Sum of Phenolic Compounds (µg/g d.w.)	DPPH (mg TE/g d.w.)	ABTS (mg TE/g d.w.)	FRAP (mg TE/g d.w.)
1	0 (−1)	5 (−1)	60 (0) (89 W)	1.84 ± 0.03	9.20 ± 0.03	747.28 ± 5.98	1.37 ± 0.02	3.02 ± 0.33	1.46 ± 0.05
2	100 (1)	5 (−1)	60 (0) (88 W)	7.12 ± 0.12	44.98 ± 0.18	896.37 ± 29.63	0.66 ± 0.04	1.82 ± 0.00	1.10 ± 0.01
3	0 (−1)	45 (1)	60 (0) (87)	10.64 ± 0.09	61.10 ± 0.13	1116.30 ± 21.78	2.06 ± 0.18	4.18 ± 0.03	2.61 ± 0.08
4	100 (1)	45 (1)	60 (0) (85)	10.52 ± 0.09	59.81 ± 0.12	1298.88 ± 21.43	1.00 ± 0.09	3.05 ± 0.00	1.81 ± 0.01
5	0 (−1)	25 (0)	20 (−1) (38 W)	1.15 ± 0.03	5.87 ± 0.04	827.16 ± 6.66	1.19 ± 0.03	1.99 ± 0.01	1.42 ± 0.05
6	100 (1)	25 (0)	20 (−1) (29 W)	4.63 ± 0.08	25.67 ± 0.11	1009.69 ± 18.90	0.61 ± 0.02	1.65 ± 0.02	1.18 ± 0.01
7	0 (−1)	25 (0)	100 (1) (149 W)	6.40 ± 0.07	33.69 ± 0.09	1072.79 ± 16.85	1.38 ± 0.01	4.16 ± 0.38	2.95 ± 0.21
8	100 (1)	25 (0)	100 (1) (126 W)	11.02 ± 0.09	58.93 ± 0.12	1489.76 ± 21.25	0.93 ± 0.10	3.17 ± 0.34	1.58 ± 0.05
9	50 (0)	5 (−1)	20 (−1) (36 W)	7.90 ± 0.09	42.94 ± 0.12	1240.36 ± 21.14	1.93 ± 0.04	3.61 ± 0.20	2.33 ± 0.03
10	50 (0)	45 (1)	20 (−1) (37 W)	7.06 ± 0.08	39.20 ± 0.11	462.23 ± 19.03	2.49 ± 0.17	4.28 ± 0.03	3.05 ± 0.12
11	50 (0)	5 (−1)	100 (1) (136 W)	8.17 ± 0.13	49.49 ± 0.19	940.24 ± 32.30	2.17 ± 0.00	4.36 ± 0.02	3.44 ± 0.14
12	50 (0)	45 (1)	100 (1) (140 W)	7.64 ± 0.12	42.74 ± 0.17	931.26 ± 29.10	1.71 ± 0.03	2.95 ± 0.28	2.36 ± 0.08
13	50 (0)	25 (0)	60 (0) (86 W)	12.63 ± 0.11	67.04 ± 0.15	1808.56 ± 26.26	2.72 ± 0.00	4.72 ± 0.22	3.49 ± 0.09
14	50 (0)	25 (0)	60 (0) (87 W)	12.32 ± 0.10	67.48 ± 0.14	1878.74 ± 23.91	2.79 ± 0.00	4.92 ± 0.18	3.58 ± 0.29
15	50 (0)	25 (0)	60 (0) (85 W)	11.96 ± 0.10	67.19 ± 0.14	1816.58 ± 23.74	2.67 ± 0.04	4.85 ± 0.07	3.54 ± 0.15

X1–3: ethanol (%), time (min) and amplitude (%). d.w.: dry weight.

**Table 3:** Estimated regression coefficients of the adjusted second-order polynomial equation and analysis of variance (ANOVA) of the model.

	Phloretin (µg/g d.w.)		Phloridzin (µg/g d.w.)		Sum of Phenolic Compounds (µg/g d.w.)		DPPH (mg TE/g d.w.)		ABTS (mg TE/g d.w.)		FRAP (mg TE/g d.w.)	
	Effect	*p* Value	Effect	*p* Value	Effect	*p* Value	Effect	*p* Value	Effect	*p* Value	Effect	*p* Value
β_0_	7.0078	0.0002 **	39.4685	0.0000 **	1002.6926	0.0001 **	1.4592	0.0001 **	3.1865	0.0001 **	2.1070	0.0000 **
Linear												
β_1_	3.3139	0.0050 *	19.0022	0.0001 **	232.7921	0.0133 *	−0.7016	0.0038 **	−0.9157	0.0062 *	−0.6934	0.0020 **
β_2_	3.8383	0.0042 **	20.4948	0.0001 **	125.9876	0.0480 *	0.3551	0.0163 *	0.6771	0.0124 *	0.5599	0.0034 **
β_3_	4.0193	0.0038 **	22.0404	0.0001 **	270.0492	0.0111 *	0.0799	0.2238	1.1331	0.0045 **	0.7087	0.0021 **
Quadratic												
β_11_	3.3347	0.0027 **	18.0086	0.0000 **	306.7966	0.0042 **	1.2519	0.0007 **	1.4344	0.0014 **	1.4025	0.0003 **
β_22_	1.4400	0.0142 *	5.4554	0.0005 **	513.1239	0.0015 **	0.2026	0.0241 *	0.3789	0.0192 *	0.3890	0.0034 **
β_33_	3.1693	0.0030 **	18.1889	0.0000 **	427.9818	0.0022 **	0.4479	0.0051 *	0.6529	0.0066 *	0.3515	0.0041 **
Crossed												
β_12_	−2.7004	0.0149 *	−18.5412	0.0001 **	16.7476	0.7054	−0.1766	0.1032	0.0368	0.7532	−0.2264	0.0352 *
β_13_	0.5730	0.2282	2.7210	0.0069 *	117.2224	0.0927	0.0615	0.4233	−0.3261	0.0858 *	−0.5643	0.0059 *
β_23_	0.1538	0.6903	−1.5058	0.0219 *	384.5743	0.0098 *	−0.5103	0.0143 *	−1.0383	0.0096 *	−0.9035	0.0023 **
R^2^ model	0.9739		0.9999		0.9706		0.9644		0.9585		0.9883	
*p* model	0.0433 *		0.0430 *		0.0198 *		0.0005 **		0.0489 *		0.0114 *	
*p* lack of fit	0.1937		0.3547		0.1657		0.1201		0.1223		0.0666	

* Significant at *p* < 0.05; ** significant at *p* < 0.005.

**Table 4:** Optimal conditions selected and the model predicted values with the obtained values expressed as the mean and the standard deviation.

Parameter		Optimal Conditions				
Ethanol (%)		50				
Time (min)		23				
Amplitude (%)		65 (90 W)				
	Phloretin	Phloridzin	Sum of phenolic compounds	DPPH	ABTS	FRAP
	(µg/g d.w.)	(µg/g d.w.)	(µg/g d.w.)	(mg TE/g d.w.)	(mg TE/g d.w.)	(mg TE/g d.w.)
Predicted value	12.44 ± 2.34	67.49 ± 1.59	1834.33 ± 269.38	2.70 ± 0.43	4.85 ± 0.72	3.55 ± 0.31
Obtained value	13.21 ± 0.25	69.12 ± 2.30	1945.54 ± 24.61	2.73 ± 0.02	4.82 ± 0.66	3.53 ± 0.58
Coefficient of variation (%)	4.22	1.69	4.16	0.59	0.51	0.58
Statistical difference	N.S.	N.S.	N.S.	N.S.	N.S.	N.S.

N.S.: non-significant.

**Table 5:** Comparison of different apple pomace extracts from different varieties obtained in the optimal conditions expressed as the average and the standard deviation.

	Granny Smith		Golden Delicious		Fuji		Gala	
	Without Seed	With Seed	Without Seed	With Seed	Without Seed	With Seed	Without Seed	With Seed
Phenolic compounds (µg/g d.w.)								
*p*-coumaric acid	25.79 ± 0.21 ^b^	32.86 ± 0.20 ^a^	13.92 ± 0.08 ^e^	13.28 ± 0.19 ^e^	16.14 ± 0.12 ^d^	17.25 ± 0.02 ^c^	17.75 ± 0.09 ^c^	17.87 ± 0.08 ^c^
Caffeoylquinic acid	75.83 ± 0.32 ^g^	24.68 ± 0.22 ^h^	526.65 ± 1.45 ^a^	367.53 ± 1.76 ^d^	224.05 ± 0.13 ^e^	105.08 ± 0.44 ^f^	377.51 ± 3.10 ^c^	452.12 ± 1.57 ^b^
Catechin	174.94 ± 0.38 ^a^	96.37 ± 0.00 ^f^	126.98 ± 0.70 ^d^	104.89 ± 0.24 ^e^	149.88 ± 0.05 ^b^	79.06 ± 1.13 ^g^	148.17 ± 0.45 ^b^	138.31 ± 0.97 ^c^
Epicatechin	30.89 ± 0.2 ^a^	25.15 ± 0.02 ^c^	14.57 ± 0.13 ^e^	13.60 ± 0.15 ^f^	12.43 ± 0.04 ^g^	12.45 ± 0.02 ^g^	19.97 ± 0.06 ^d^	26.50 ± 0.32 ^b^
Procyanidin dimer	164.82 ± 0.54 ^a^	95.51 ± 0.23 ^b^	93.13 ± 0.18 ^b^	55.80 ± 0.23 ^e^	61.68 ± 0.86 ^d^	41.47 ± 0.96 ^f^	72.31 ± 0.70 ^b^	71.42 ± 0.92 ^b^
Procyanidin trimer	61.63 ± 0.79 ^a^	38.50 ± 0.11 ^b^	24.59 ± 0.13 ^e^	16.19 ± 0.11 ^f^	23.83 ± 0.48 ^e^	16.14 ± 0.46 ^f^	28.41 ± 0.31 ^c^	29.24 ± 1.17 ^d^
Phloretin-2′-O-xyloglucoside	70.23 ± 0.48 ^a^	40.37 ± 1.14 ^d^	54.97 ± 0.14 ^c^	36.19 ± 0.34 ^e^	3.95 ± 0.21 ^f^	< LOQ	51.56 ± 0.79 ^c^	60.93 ± 0.83 ^b^
Quercetin-3-O-galactoside	60.54 ± 0.71 ^e^	137.50 ± 2.11 ^b^	37.16 ± 1.40 ^f^	55.30 ± 1.69 ^e^	106.88 ± 0.17 ^d^	145.45 ± 2.51 ^b^	124.97 ± 0.09 ^c^	209.40 ± 2.77 ^a^
Quercetin-3-O-glucoside	<LOQ	153.65 ± 3.99 ^a^	<LOQ	<LOQ	39.12 ± 0.05 ^d^	90.83 ± 2.44 ^c^	24.88 ± 0.17 ^e^	107.90 ± 1.29 ^b^
Rutin	<LOQ	131.32 ± 3.29 ^b^	<LOQ	<LOQ	67.03 ± 0.86 ^c^	150.67 ± 1.97 ^a^	<LOQ	38.81 ± 0.77 ^d^
Phloretin	38.89 ± 0.35 ^h^	64.71 ± 0.16 ^f^	84.41 ± 0.08 ^e^	115.63 ± 0.11 ^a^	97.89 ± 0.00 ^c^	106.13 ± 0.18 ^b^	43.12 ± 0.15 ^g^	95.96 ± 0.07 ^d^
Phloridzin	30.43 ± 0.25 ^f^	92.95 ± 1.16 ^e^	139.13 ± 1.46 ^d^	263.38 ± 3.75 ^a^	202.92 ± 1.47 ^b,c^	211.35 ± 0.12 ^b^	39.15 ± 0.30 ^f^	198.93 ± 1.02 ^c^
Quercetin-3-O-arabinopyranoside	39.97 ± 0.38 ^f^	111.15 ± 2.85 ^c^	11.95 ± 0.11 ^g^	35.17 ± 1.35 ^f^	90.53 ± 0.60 ^d^	141.98 ± 2.28 ^a^	75.37 ± 1.07 ^e^	123.29 ± 2.44 ^b^
Quercetin-3-O-arabinofuranoside	<LOQ	<LOQ	<LOQ	<LOQ	<LOQ	7.14 ± 0.00	<LOQ	<LOQ
Quercetin-3-O-xylanoside	<LOQ	<LOQ	<LOQ	<LOQ	<LOQ	<LOQ	<LOQ	<LOQ
Isorhamnetin-3-O-glucoside	<LOQ	<LOQ	<LOQ	<LOQ	<LOQ	<LOQ	<LOQ	<LOQ
Quercetin-3-O-rhamnoside	73.41 ± 0.53 ^e^	124.32 ± 2.24 ^c^	50.00 ± 0.15 ^f^	96.33 ± 1.47 ^d^	103.66 ± 0.07 ^d^	175.99 ± 2.38 ^a^	101.99 ± 0.88 ^d^	153.79 ± 1.55 ^b^
Sum of phenolic compounds	922.39 ± 6.43 ^e^	1169.04 ± 17.01 ^c,d^	1177.46 ± 6.00 ^c,d^	1177.76 ± 11.39 ^c,d^	1199.98 ± 5.10 ^c^	1297.41 ± 14.91 ^b^	1125.14 ± 8.15 ^d^	1724.47 ± 15.77 ^a^
Amygdalin (µg/g d.w.)	n.d.	17.48 ± 0.74 ^c^	n.d.	60.07 ± 0.28 ^a^	n.d.	48.32 ± 0.26 ^b^	n.d.	13.14 ± 0.13 ^d^
Antioxidant assays (mg TE/g d.w.)								
DPPH	5.11 ± 0.34 ^a,b,c^	5.47 ± 0.06 ^a,b^	3.89 ± 0.03 ^b,c^	4.65 ± 0.30 ^c^	5.23 ± 0.46 ^a,b^	6.10 ± 0.20 ^a^	5.00 ± 0.49 ^a,b,c^	6.26 ± 0.38 ^a^
ABTS	11.28 ± 0.29 ^a,b,c^	12.68 ± 0.25 ^a^	7.72 ± 0.26 ^d^	8.54 ± 0.46 ^d^	9.43 ± 0.93 ^c,d^	10.76 ± 0.61 ^a,b,c^	10.57 ± 0.28 ^b,c^	11.94 ± 0.54 ^a,b^
FRAP	7.74 ± 0.74 ^a,b^	8.26 ± 0.02 ^a^	4.00 ± 0.45 ^c^	4.78 ± 0.00 ^b,c^	6.17 ± 0.69 ^a,b,c^	7.22 ± 1.07 ^a,b^	6.74 ± 0.33 ^a,b,c^	8.37 ± 0.52 ^a^

n.d.: non-detected; LOQ: limit of quantification. Letters ^a–h^ indicate significant differences (*p* < 0.05).

## Data Availability

Data is contained within the article or supplementary material.

## Supplementary Materials

The following supporting information can be downloaded at: https://www.mdpi.com/article/10.3390/foods11233809/s1, Table S1: Identified amygdalin by HPLC–ESI–TOF–MS in apple pomace with seed extracts; Figure S1: Negative-mode product ion spectrum of amygdalin (465 *m*/*z*); Figure S2: HPLC–TOF–MS chromatogram of apple pomace with seed extracts; Figure S3: Pearson’s correlation heatmap for all the analyses performed in the different varieties of apple pomace analyzed with and without seed.
