# Supplementary material for: Establishment of a Sonotrode Ultrasound-Assisted Extraction of Phenolic Compounds from Apple Pomace
Source: Foods. 2022 Nov 25;11(23):3809. doi: 10.3390/foods11233809 (PMC9740410; doi:10.3390/foods11233809)
Supplement: Supplementary file 1 [file foods-11-03809-s001.zip › foods-2017955-supplementary.pdf]

Supplementary Materials

Table S1. Identified amygdalin by HPLC-ESI-TOF-MS in apple pomace with seed extracts.

Peak	Rt (min)	Observed *m/z*	Calculated *m/z*	Error (ppm)	Score (%)	Molecular formula	In source *m/z* fragments	Compound name
1	3.47	456.1504	456.1506	-0.4	94.30	$C_{20}H_{26}NO_{11}$	323.0913	Amygdalin

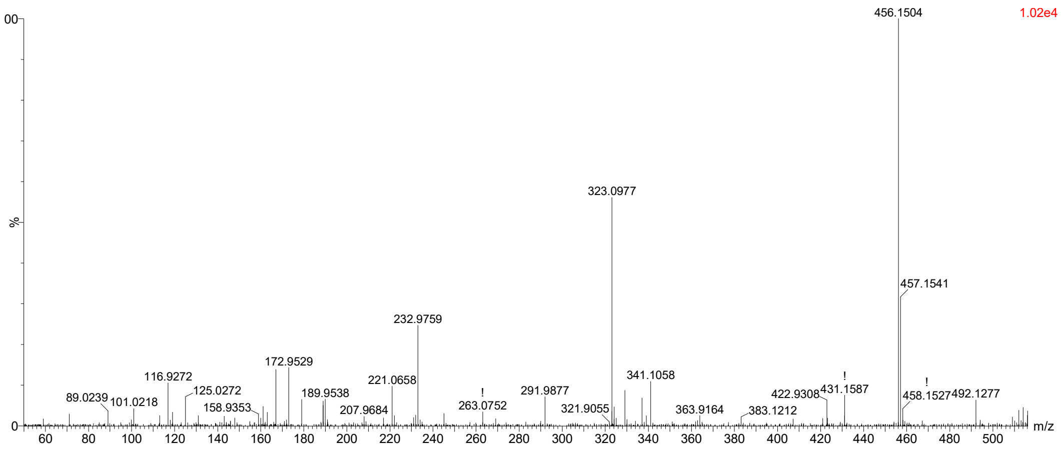

Figure S1. Negative mode product ion spectrum of amygdalin (465 m/z).

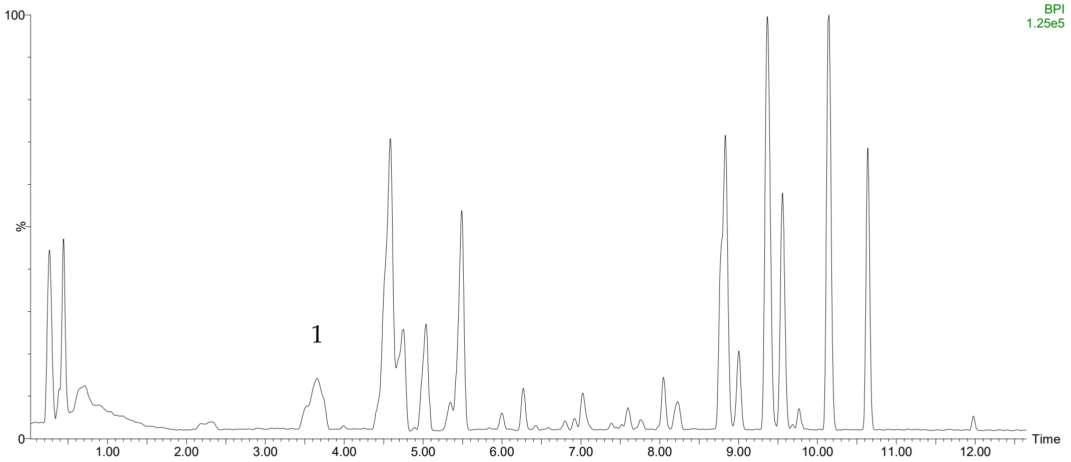

Figure S2. HPLC-TOF-MS chromatogram of apple pomace with seed extracts. 1: Amygdalin.

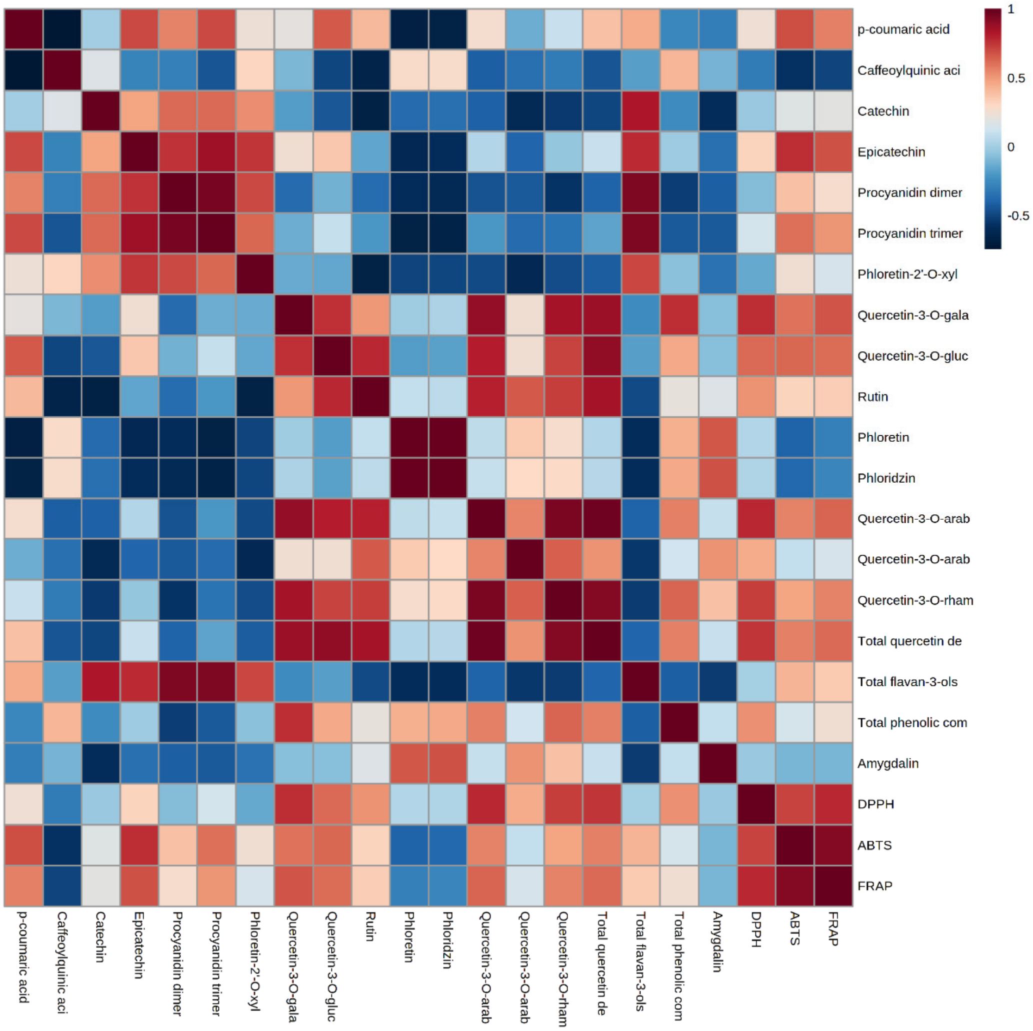

Figure S3. Pearson's correlation heatmap for all the analyses performed in the different varieties of apple pomace analyzed with and without seed. .
